# Obituary

**DOI:** 10.3402/jev.v3.23842

**Published:** 2014-02-03

**Authors:** F. Hochberg, C. Gardiner, Y.S. Gho, D. Gupta, A. Hill, J. Lötvall, P. Quesenberry, L. Rajendran, J. Rak, H. Tahara, D. Taylor, C. Théry, M. Wauben

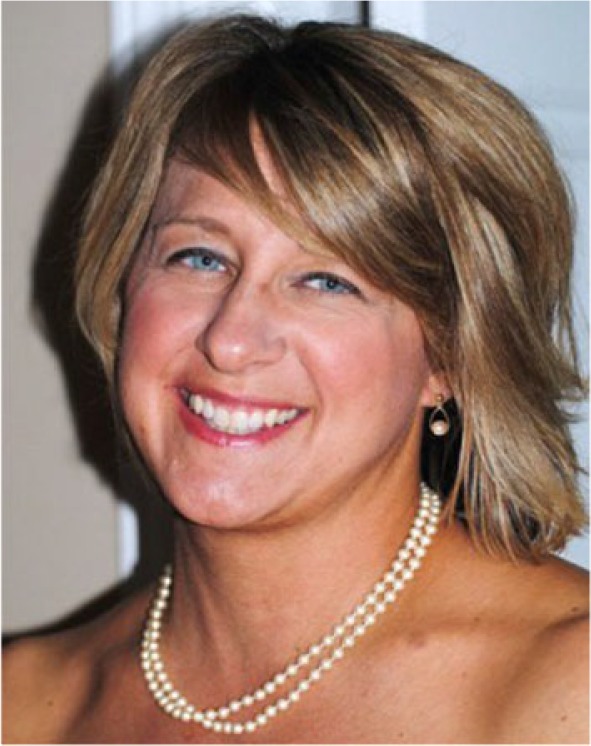

Dr. Melissa Piper

It is with much sadness that the Executive Board of ISEV announces the death of one of its members, Dr. Melissa “Missy” Piper, aged 45, who succumbed to complications of colon cancer on Wednesday, 11 December 2013. She is survived by her husband, Joseph Klusacek; daughter, Megan Hunter; son, Matthew Hunter; step-daughter, Allison Klusacek; step-son, Adam Klusacek; and her parents, Victor S. Piper Sr. and Helen Piper, and brother, Victor S. Piper Jr.

Dr. Piper grew up in Latrobe, PA, and graduated from Indiana University of Pennsylvania. She trained at the National Institutes of Health and received her PhD from The Ohio State University. Her doctorate and post-doctorate work on colony-stimulating factors was in Hematology–Oncology but, on joining the laboratory of Clay Marsh, she began a transformation to microvesicular miRNA studies as applied to respiratory diseases. Dr. Marsh notes: “We were a macrophage cell biology and signaling lab before Melissa joined. I knew she was very bright, very talented technically and I attended a conference she taught and was very impressed. I talked with her and asked if she was willing to take another challenge and help me transition the lab to do more mechanistic work using molecular biology tools/techniques and to perform epigenetic work with classical methods to detect methylation and histone modification.” She facilitated molecular techniques in *in-vitro* work and moved into genetically modified cre-lox mouse models back bred to purity and introduced a new tomato red reporter system. This switch, Dr. Marsh adds, “was transformational for us.” From a devotion to research on extracellular vesicles called microvesicles (MV), Melissa provided in 2008 and 2013, two seminal observations which serve as hallmarks in the work of the ISEV community.

Providing the history of her 2008 article (Detection of microRNA expression in human peripheral blood microvesicles. PLoS ONE. Vol. 3, no. 11: e3694 (November 2008)), Dr. Marsh writes: “This paper was evidence of Melissa's relentless search for answers. We found a signal in cellular supernatants that was resistant to DNAse, RNAse and heat but not freeze/thaw. We surmised that perhaps miRNA in the MV could be responsible and isolated MV from the supernatants. We found MV with miRNA. Knowing that these MV were reported in disease, we next wondered if these MV could circulate in healthy people and, if so, if they also contained miRNA. We surmised that this may be a more foundational communication system between organs. This was even more likely when we did the Ingenuity Pathway assessment on miRNA in the plasma MV and found that they were predicted to control bone marrow events.”

Young investigator members of ISEV will benefit from studying the legacy of Melissa. Her 2008 paper asked a fundamental question: “How do blood cells communicate?”. As a hematology researcher, soon to expand into systemic cancers, she reviewed the connection between miR-15 and miR-16 and B-cell chronic lymphocytic leukaemia and initiation and progression of numerous cancers: “Recent evidence reveals that genetic exchange of mRNA and miRNA between cells can be accomplished through microvesicles, or exosome-mediated transfer.” Her profiles of miRNA within microvesicles from plasma, platelets and mononuclear phagocytes from healthy donors anticipated the debate over speciation of these vesicles as well as the NIH Common Fund commitment to determine the “normalized” pattern of vesicle production. She hypothesized a role for MV in cell signaling, hematopoiesis, cell differentiation and metabolism: “the detection of tissue specific miRNAs and microvesicles in the peripheral blood may be a frequent event upon tissue damage. We predict that the plasma microvesicles may be selective in their target cells … each subpopulation of the plasma microvesicles and their miRNAs will be important factors in the regulation of immune responses and hematopoiesis.” With Leni Moldovan, she developed novel methodologies of purification, quantification and subpopulation purification that were taken to provisional patents as were her techniques for isolation of the content of circulating MV in normal individuals. She understood the diagnostic and therapeutic implications of her work.

Melissa's focus soon moved to macrophages (Macrophage microvesicles induce macrophage differentiation and miR-223 transfer. Blood, 121: 984–995 (February 2013)) in lung disease and pulmonary fibrosis. She had found that RNA molecules contained in the macrophage-derived microvesicles were transported to target cells, including monocytes, endothelial cells, epithelial cells and fibroblasts. Furthermore, *miR-223* was transported to target cells and “we hypothesize that microvesicles bind to and activate target cells. The present paper is the first to demonstrate that macrophage-derived microvesicles induced cellular differentiation in target cells.” To understand pulmonary fibrosis, she believed that miRNA-regulated epigenetic elements (DNMTs, HDACs, histone methyltransferases) are likely important as are miRNA in aged individuals.

Melissa joined the ISEV Board in 2012. In submitting her application to ISEV she indicated: “The focus of my research involves understanding the role of extracellular vesicles (EVs) and microRNA in survival and function of innate immune cells as well as their maturation under normal homeostasis. We predict that specific subpopulations of EVs in the peripheral blood may increase in certain diseases as well as have altered content. Currently in the scientific community, there is a lack of understanding of what defines an EV and the importance of these in the immune response. This has led to difficulty in publishing our studies in high impact journals and funding. As an ISEV board member, I will focus on the education of the scientific community and standardization of nomenclature. I would also promote an initiative for the establishment of an ISEV peer reviewed journal for investigators to publish and further this area of research.” Melissa realized these goals and was an active member of the ISEV Board and community, contributing to the annual ISEV meetings in 2012 and 2013, in addition to participating in the first ISEV workshop on Extracellular Vesicle RNA held in NYC in October 2012. Her contributions to these meetings were instrumental in providing chapters for the ISEV Journal's position paper on Standardization of Sample Collection (authored by Dr. K. Witwer and colleagues, J Extracell Vesicles 2: 20360 (May 2013)) and for the report on ISEV-2013 Boston meeting (authored by Dr. E. Aikawa and colleagues, J Extracell Vesicles 2: 23070 (December 2013)). Those who served with her remember her inquisitive mind, her concern about nurturing young investigators and her striving to improve how science is communicated. She had trained over twenty early scientists who recall her as a “loyal, warm, and generous friend and colleague. During her time within Ohio State University, in the Division of Pulmonary, Allergy, Critical Care & Sleep Medicine, she, Melissa, touched countless lives, serving as a giving mentor to junior scientists and physician trainees.” She directed the animal core and the translational core of her division and opened these resources to all. She worked tirelessly to serve others and sought excellence in everything she did. She was organized, attentive to detail and displayed a thirst to help others. She would stay up to 4 AM working on grants and was “dedicated to her profession.” Peter Mohler (Director – Dorothy M. Davis Heart & Lung Research Institute) of her institution wrote of her: “Thinking of Melissa reminds me to focus on what really matters and in turn, this Emerson quote reflects her legacy to us: To laugh often and much; To win the respect of intelligent people and the affection of children; To earn the appreciation of honest critics and endure the betrayal of false friends; To appreciate beauty, to find the best in others; To leave the world a bit better, whether by a healthy child, a garden patch, or a redeemed social condition; To know even one life has breathed easier because you have lived. This is to have succeeded.”

As a mother and a friend she spent time with her family; grants and research began when everyone went to sleep. She was a competitive runner and loved the beaches of Florida. We at ISEV met Melissa as a colleague in “The New Science” of vesicles and lost her as a friend. All who knew her, especially her family, should be aware of the passion with which she did everything, thought of her work and planned for the future. This passion touched the way in which we communicate science, how we joined together at our meetings and how we supported the intellect of young investigators. She thought of herself as a “wise young investigator” in a field with a short history and a bright future. She was extremely motivating and came up with brilliant ideas during discussions. She brought to us a template for explaining to students their role in creativity. She was not a wilting flower; she stuck to her guns, fought for her work and her funding. She was strong, well-spoken and a great listener. Her commitment never wavered. She fought for every step, every progress. While she was dying, she apologized for ceasing her scientific activities for ISEV: “I am sorry to inform you but I can no longer participate in this review. My health has deteriorated and I am entering Hospice.” All of us in the scientific family of ISEV lost a friend only two days after having received that message.

The Ohio State University has created a new award in Melissa's honour for notable achievement by a junior faculty member.

*F*. *Hochberg*, *C*. *Gardiner*, *Y.S*. *Gho*, *D*. *Gupta*, *A*. *Hill*, *J*. *Lötvall*, *P*. *Quesenberry*, *L*. *Rajendran*, *J*. *Rak*, *H*. *Tahara*, *D*. *Taylor*, *C*. *Théry*, *M*. *Wauben*.

